# SpxA1 Involved in Hydrogen Peroxide Production, Stress Tolerance and Endocarditis Virulence in *Streptococcus sanguinis*

**DOI:** 10.1371/journal.pone.0040034

**Published:** 2012-06-29

**Authors:** Lei Chen, Xiuchun Ge, Xiaojing Wang, Jenishkumar R. Patel, Ping Xu

**Affiliations:** The Philips Institute of Oral and Craniofacial Molecular Biology, Virginia Commonwealth University, Richmond, Virginia, United States of America; University of Kansas Medical Center, United States of America

## Abstract

*Streptococcus sanguinis* is one of the most common agents of infective endocarditis. Spx proteins are a group of global regulators that negatively or positively control global transcription initiation. In this study, we characterized the *spxA1* gene in *S. sanguinis* SK36. The *spxA1* null mutant displayed opaque colony morphology, reduced hydrogen peroxide (H_2_O_2_) production, and reduced antagonistic activity against *Streptococcus mutans* UA159 relative to the wild type strain. The Δ*spxA1* mutant also demonstrated decreased tolerance to high temperature, acidic and oxidative stresses. Further analysis revealed that Δ*spxA1* also exhibited a ∼5-fold reduction in competitiveness in an animal model of endocarditis. Microarray studies indicated that expression of several oxidative stress genes was downregulated in the Δ*spxA1* mutant. The expression of *spxB* and *nox* was significantly decreased in the Δ*spxA1* mutant compared with the wild type. These results indicate that *spxA1* plays a major role in H_2_O_2_ production, stress tolerance and endocarditis virulence in *S. sanguinis* SK36. The second *spx* gene, *spxA2,* was also found in *S. sanguinis* SK36. The *spxA2* null mutant was found to be defective for growth under normal conditions and showed sensitivity to high temperature, acidic and oxidative stresses.

## Introduction

*Streptococcus sanguinis* is a member of the human indigenous oral microbiota and is known as a pioneering colonizer in the formation of dental plaque [1]–[4]. *S. sanguinis* is also one of the most common agents of infective endocarditis (IE) among the viridans streptococci [5]–[7]. IE is a serious endovascular infection that carries a high risk of morbidity and mortality and is the fourth leading cause of life-threatening infectious disease syndromes [8]. In cases of IE, it is thought that damage to the heart results in the formation of sterile cardiac “vegetations” composed of platelets and fibrin. These sterile vegetations can then be colonized by certain bacteria during periods of bacteremia [9]. This view is supported by animal studies in which formation of sterile vegetation by cardiac catheterization is required for the efficient establishment of streptococcal endocarditis [10].

On the other hand, in the oral cavity, *S. sanguinis* is antagonistic against *S. mutans*, a facultative anaerobic bacterium that is a significant contributor to tooth decay [11], [12]. It has been reported that relatively high proportions of *S. sanguinis* are generally found in dental plaque in association with lower levels of *S. mutans* [12], [13]. This antagonistic activity against *S. mutans* is reported to be via hydrogen peroxide (H_2_O_2_) production by *S. sanguinis* [14]. So the production of H_2_O_2_ is considered an important property of *S. sanguinis* concerning its protective role in the oral community.

Spx proteins are a group of global regulators that interact directly with the α-subunit of the RNA polymerase (RNAP) and thereby, negatively or positively control global transcription initiation [15], [16]. The Spx global regulator is highly conserved among low-GC Gram-positive bacteria [16]. It has been well studied in *Bacillus subtilis* [17]–[19] and global analysis has shown that it regulates the expression of different subsets of genes involved in oxidative stress, developmental programs and energy-consuming growth-related functions [15]. It is reported that Spx is a substrate of ClpXP proteolysis [19], which is critical for maintaining cellular homeostasis as well as expression of virulence properties, and that its accumulation is responsible for the pleiotropic phenotypes associated with *clpXP* mutations [20]. The interactions between ClpXP and Spx are suggested to be relatively conserved among Gram-positive bacteria [21]. To date, Spx regulators have been studied in many species, including *Lactococcus lactis* [22], *B. subtilis* [15], [17], [20], *S. mutans* [23] and *Streptococcus pneumoniae* [24], where they fulfill important roles in general stress protection. Concerning the mechanism of regulation, Spx has been shown to be involved in transcriptional repression by interacting with the C-terminal domain of the RNAP α-subunit (α-CTD), which may prevent interaction with specific activator proteins [15]. Furthermore, activation of transcription requires contact between the Spx/RNA polymerase complex and upstream promoter DNA, thereby allowing Spx-induced engagement of RNA polymerase subunits with the core promoter [25].

Due to its important regulatory role, Spx is involved in various physiological functions. In *Staphylococcus aureus*, Spx was shown a global effector impacting stress tolerance and biofilm formation [26]. SpxA1 was shown to be involved in X-state (competence) development in *S. pneumoniae* [24] and two Spx proteins were identified with the ability to modulate stress tolerance, survival and virulence in dental caries in *S. mutans* [23].

Here we report on the identification of a *spxA1* gene in *S. sanguinis* SK36. Subsequent functional characterization of *spxA1* revealed that *spxA1* is involved in H_2_O_2_ production, stress tolerance and IE virulence in *S. sanguinis* SK36. Preliminary characterization of *spxA2*, which encodes the second Spx protein, is also reported.

## Results

### Δ*spxA1* Demonstrated Opaque Colony Morphology and a Reduced Rate of H_2_O_2_ Production

**Figure 1:**
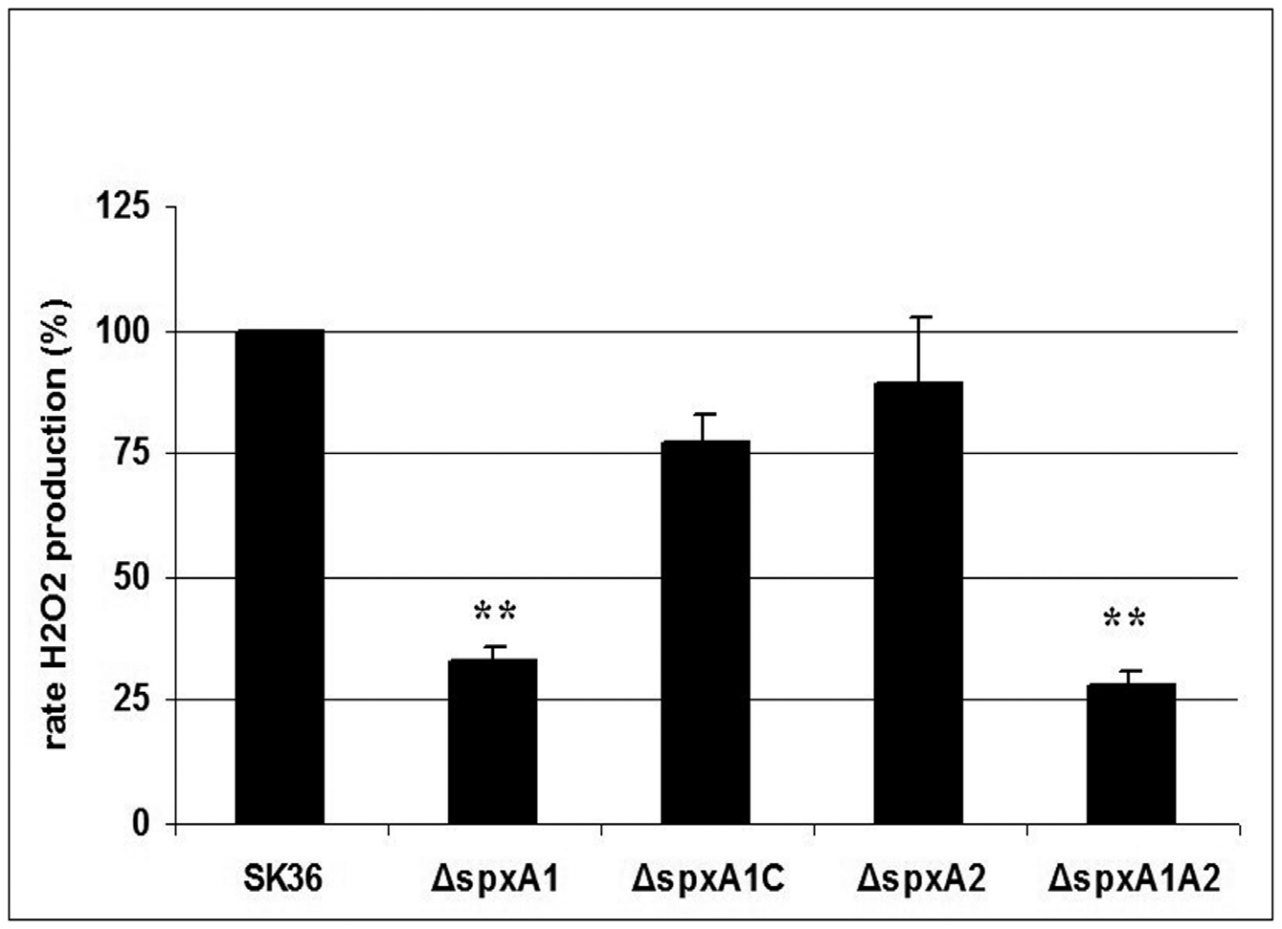
H_2_O_2_ production in *S. sanguinis* strains. H_2_O_2_ production normalized to culture densities was determined relative to that produced by the wild type strain SK36. Results are expressed as the mean of percentage values relative to the wild-type strain SK36 from three biological repeats. Statistical significance is indicated (**P<0.01).

During genome-wide gene deletion studies in *S. sanguinis* SK36 [27], we identified a mutant of SSA_0937, (denoted as Δ*spxA1*) that demonstrated opaque colonies when cultured on BHI plates. Opaque colony morphology was previously found to correlate with decreased H_2_O_2_ production in *S. sanguinis* SK36 [28]. Subsequent quantification of H_2_O_2_ production in this mutant showed that when compared with the SK36 wild type strain, H_2_O_2_ levels in Δ*spxA1* were significantly reduced. Specifically, the mutant produced only ∼33% of the H_2_O_2_ levels observed in the wild type (Fig. 1). These results suggest that SxpA1 is involved in colony morphology and H_2_O_2_ production in *S. sanguinis* SK36.

**Figure 2:**
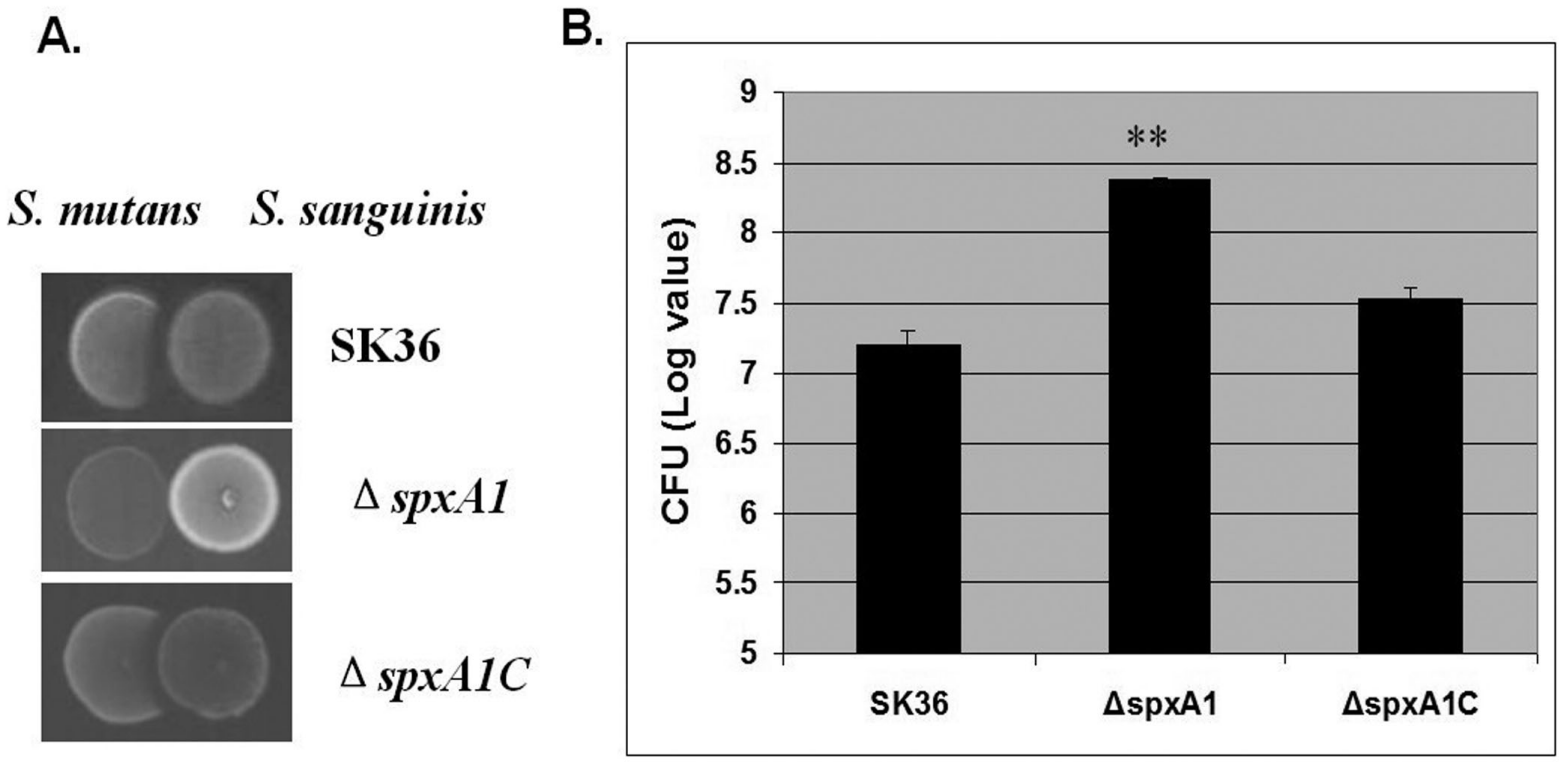
Inhibitory effect of *S. sanguinis* strains on *S. mutans* (A) Inhibition assay on plates. Overnight cultures of *S. sanguinis* SK36, *ΔspxA1* or *ΔspxA1C* were inoculated on BHI plates, which were incubated for 16 h at 37°C under microaerobic conditions. *S. mutans* UA159 was then inoculated next to the pioneer colonizer, and the plates were further incubated overnight and photographed. (B) Inhibition assay in liquid media. Overnight cultures of *S. sanguinis* SK36, *ΔspxA1* or *ΔspxA1C* were adjusted to the same optical density and mixed with the *S. mutans smx_42* in fresh BHI medium. After overnight growth, the cells were serially diluted and plated on BHI plates supplemented with chloramphenicol. The CFUs of *S. mutans* are indicated in logarithmic values, with standard deviations calculated from triplicate mixtures (**P<0.01 relative to the values obtained for the SK36 mixture).

Since the formation of H_2_O_2_ in *S. sanguinis* plays an important role in interspecies interactions within the oral microflora [14], we performed competition assays [28] to examine whether Δ*spxA1* showed any difference from the parent strain, SK36, with regard to *S. sanguinis*’ capacity for antagonism against *S. mutans*. The results indicated that Δ*spxA1* showed reduced antagonistic activity against *S. mutans* UA159 both on plates (Fig. 2A) and in broth media (Fig. 2B), suggesting SpxA1 is an important protein that confers a competitive advantage for *S. sanguinis*.

To ensure the involvement of *spxA1* in H_2_O_2_ production, we next constructed a complemented strain whereby *spxA1* was re-introduced back into the mutant (Δ*spxA1*). For this purpose, a chloramphenicol resistance cassette was placed downstream of *spxA1* for selection. The resulting complemented strain, denoted as Δ*spxA1C*, was examined for colony morphology and H_2_O_2_ production. The results indicated that the morphology of Δ*spxA1C* was restored to semi-transparent similar to the wild type and that H_2_O_2_ levels were also partially restored (∼77% that of the wild type; Fig. 1). These data confirm the involvement of *spxA1* in H_2_O_2_ production, demonstrating that the observed phenotypes associated with Δ*spxA1* were not the result of polar effects.

### Sequence Analysis of SpxA1 and Identification of a Second Spx in *S. sanguinis*

**Figure 3:**
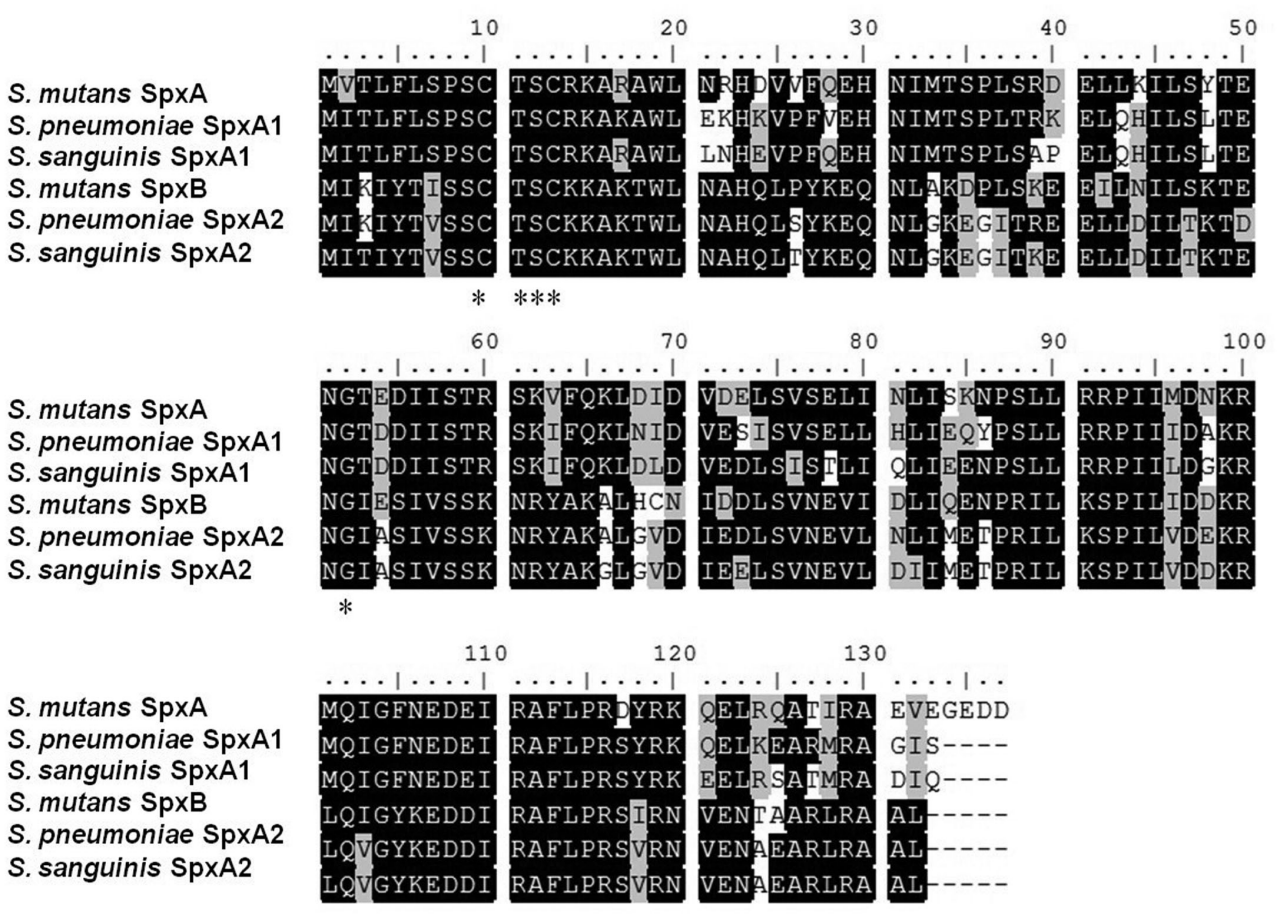
Alignment of amino acid sequences of the two Spx proteins from *S. mutans*, *S. pneumonia* and *S. sanguinis*. Identical residues are shaded in black and similar ones in gray. The conserved CXXC motif and Gly_52_ residue discussed in the text are stars (*) labeled. The GenBank accession numbers: *S. mutans* SpxA, SMU.1142c; *S. pneumonia* SpxA1, SPR1262; *S. sanguinis* SpxA1, SSA_0937; *S. mutans* SpxB, SMU.2084c; *S. pneumonia* SpxA2, SPR0173; *S. sanguinis* SpxA2, SSA_2244.

SpxA1 consists of 133 amino acids and is a member of the arsenate reductase family, which includes true arsenate reductases (ArsC) and Spx proteins as revealed by *in silico* analyses. Spx proteins are a group of global regulators that interact directly with the RNAP and have been well studied in *B. subtilis* [17]–[19] and streptococci species including *S. pneumoniae* [24] and *S. mutans* [23]. Motif analysis indicated that SpxA1 possesses the conserved amino terminus motif Cys_10_-X-X-Cys_13_ (CXXC) (Fig. 3) that has previously been shown to sense the intracellular redox state via disulfide bond formation [29], [30]. Moreover, the Gly_52_ residue that is essential for the interaction of the *B. subtilis* SpxA with the RNAP α-CTD [30]–[32] is also conserved in SpxA1 (Fig. 3). These findings suggest that SpxA1 in *S. sanguinis* may also share important physiological functions common to other Spx proteins.

Two Spx paralogs have previously been identified in *S. pneumoniae* and *S. mutans* [23], [24], therefore, we performed a BLASTP search against proteins annotated in the genome of *S. sanguinis* SK36 [4] to determine if paralogs were also present in this strain. Using the SpxA (SMU1142) and SpxB (SMU2084) of *S. mutans* UA159 [23], two significant hits were identified; one of which was SSA_0937 (SpxA) and the other was SSA_2244 (Fig. 3). Since SSA_2244 is approximately the same size (132 AA) as SpxA1 and also contains the conserved amino terminus motif CXXC and the Gly_52_ residue, we named this second protein, SpxA2.

Next, SpxA2 was inactivated by replacing the ORF (SSA_2244) with the kanamycin resistance cassette (*aphA-3*) to determine its function in *S. sanguinis* SK36 [33]. We also constructed a simultaneous deletion of *spxA1* and *spxA2* to determine its function in relation to *spxA1*. In *S. pneumoniae R6*, it was reported that *spxA1* and *spxA2* were essential since simultaneous inactivation of both genes was lethal [24]. While in *S. mutans* UA159, the double mutant of the two *spx* genes was viable [23]. We were able to successfully obtain the simultaneous double mutant (Δ*spxA1A2*), indicating that, similar to *S. mutans* UA159, *spxA1* and *spxA2* are not essential in *S. sanguinis* SK36. Phenotypic analysis showed that the rate of H_2_O_2_ production of Δ*spxA2* was not significantly different from wild type (Fig. 1), consistent with its normal semi-transparent colony morphology. However, the double mutant Δ*spxA1A2* showed reduced rates of H_2_O_2_ production (Fig. 1).

### SpxA1 Involved in Tolerance to High Temperature, Reduced pH and Oxidative Stresses

**Figure 4:**
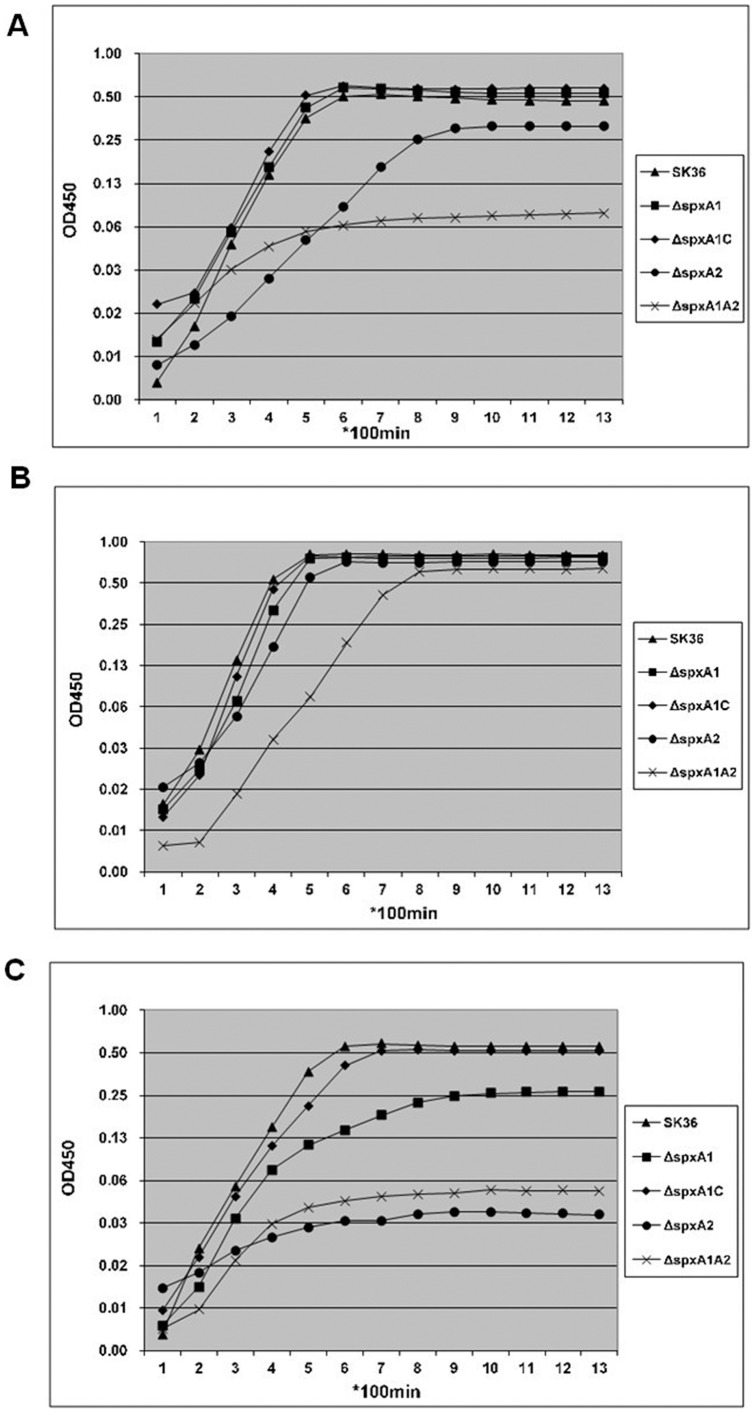
Growth curves of *S. sanguinis* strains. Overnight cultured bacteria were trans-inoculated into wells in a 96-well plate containing 200 µl BHI media with 1% inoculation and OD_450_ of each well was determined with a FLUOstar plate reader every 100 minutes at 37°C (A) or 40°C (C) under aerobic condition. For anaerobic conditions (B), an overlay of 50 µl of sterile mineral oil was included in each well of the plate to maintain an anaerobic environment. The growth curves were obtained from average OD_450_ of at least three repeated wells.

Since Spx proteins were found to play important roles in general stress protection in many species [23], [24], [26], we wondered if this too, was the case in *S. sanguinis* SK36. First, the impact of Spx proteins was analyzed in response to high temperature stress. Wild type and mutant strains were both cultured under a normal growth temperature of 37°C or a higher growth temperature of 40°C (Fig. 4). The wild type strain was able to grow under both temperature conditions (37°C and 40°C). While Δ*spxA1* did not show any significant difference in growth compared to the wild type when cultivated under the normal growth temperature (37°C) either aerobically (Fig. 4A) or anaerobically (Fig. 4B), when cultivated at a higher temperature (40°C) (Fig. 4C), the growth of the Δ*spxA1* mutant was significantly compromised compared to the wild type, as well as compared to the growth at 37°C (Fig. 4A). As expected, the growth of the complemented strain (Δ*spxA1C)* under a higher temperature was restored to that of the wild type (Fig. 4C). This indicated that *spxA1* was involved in tolerance to high temperature stress in *S. sanguinis* SK36. At the same time, another *spx* mutant, Δ*spxA2* was defective in growth both when cultivated under aerobic (Fig. 4A) or higher temperature conditions (Fig. 4C). The double mutant Δ*spxA1A2* was much slower in growth than Δ*spxA2* under aerobic (Fig. 4A) or higher temperature conditions (Fig. 4C).

**Figure 5:**
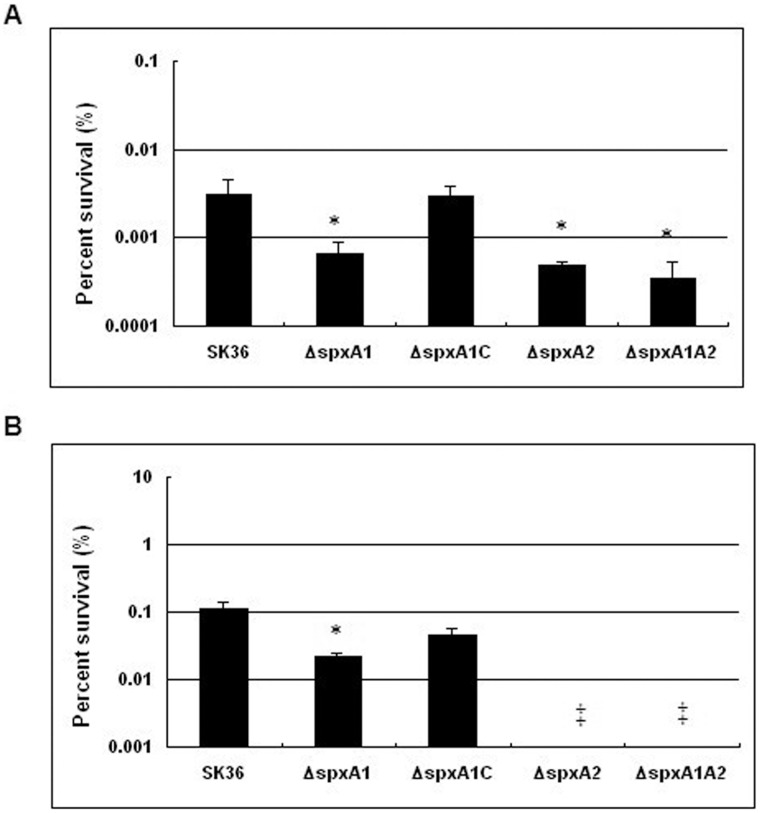
Acid tolerance (A) and H_2_O_2_ sensitivity (B) assays of *S. sanguinis* strains. The survival percentage after treatment was presented. Data from three biological replicates were averaged and the statistical significance differences relative to SK36 were determined. (*P<0.05). ‡: Bacteria did not survive.

Next, acid tolerance assays were performed. Results demonstrated that Δ*spxA1* showed a reduced ability to survive under acidic stress compared with the wild type SK36 strain (Fig. 5A). The acidic tolerance of the complemented strain Δ*spxA1C* was also analyzed and results demonstrated that its tolerance to acid was restored (Fig. 5A). These results suggest that SpxA1 plays a role in acidic stress tolerance. In the case of the Δ*spxA2* mutant and the double mutant Δ*spxA1A2*, both mutants demonstrated reductions in their ability to survive acidic stress (Fig. 5A). In addition to *spxA1*, these data indicate that *spxA2* may also play an important role in acidic stress tolerance.

Finally, we investigated the role of *spx* genes in H_2_O_2_ protection using H_2_O_2_ sensitivity assays. The results showed that as expected, Δ*spxA1* was sensitive to H_2_O_2_ killing (Fig. 5B) relative to the wild type SK36 strain; while H_2_O_2_ sensitivity was partially restored in the complemented strain *ΔspxA1C* (Fig. 5B). To further address the possible polar effect, we inactivated two of the *spxA1* downstream genes, *ssa_0938* and *ssa_0939*, respectively and checked their H_2_O_2_ sensitivity under the same condition. Both mutants showed no significant difference from the wild type, which further supported the H_2_O_2_ sensitivity role of *spxA1* (data not shown). The Δ*spxA2* mutant and the double mutant Δ*spxA1A2* also demonstrated an increase in sensitivity to oxidative stress (Fig. 5B); these strains were unable to survive at concentrations as low as 20 mM H_2_O_2_ condition for 60 min (Fig. 5B). Taken together, these results confirm that *spxA1* and *spxA2* play significant roles in oxidative stress tolerance of *S. sanguinis* SK36.

### *ΔspxA1* Demonstrated Reduced Competitiveness in CI Assays in the Animal Model of IE

**Figure 6:**
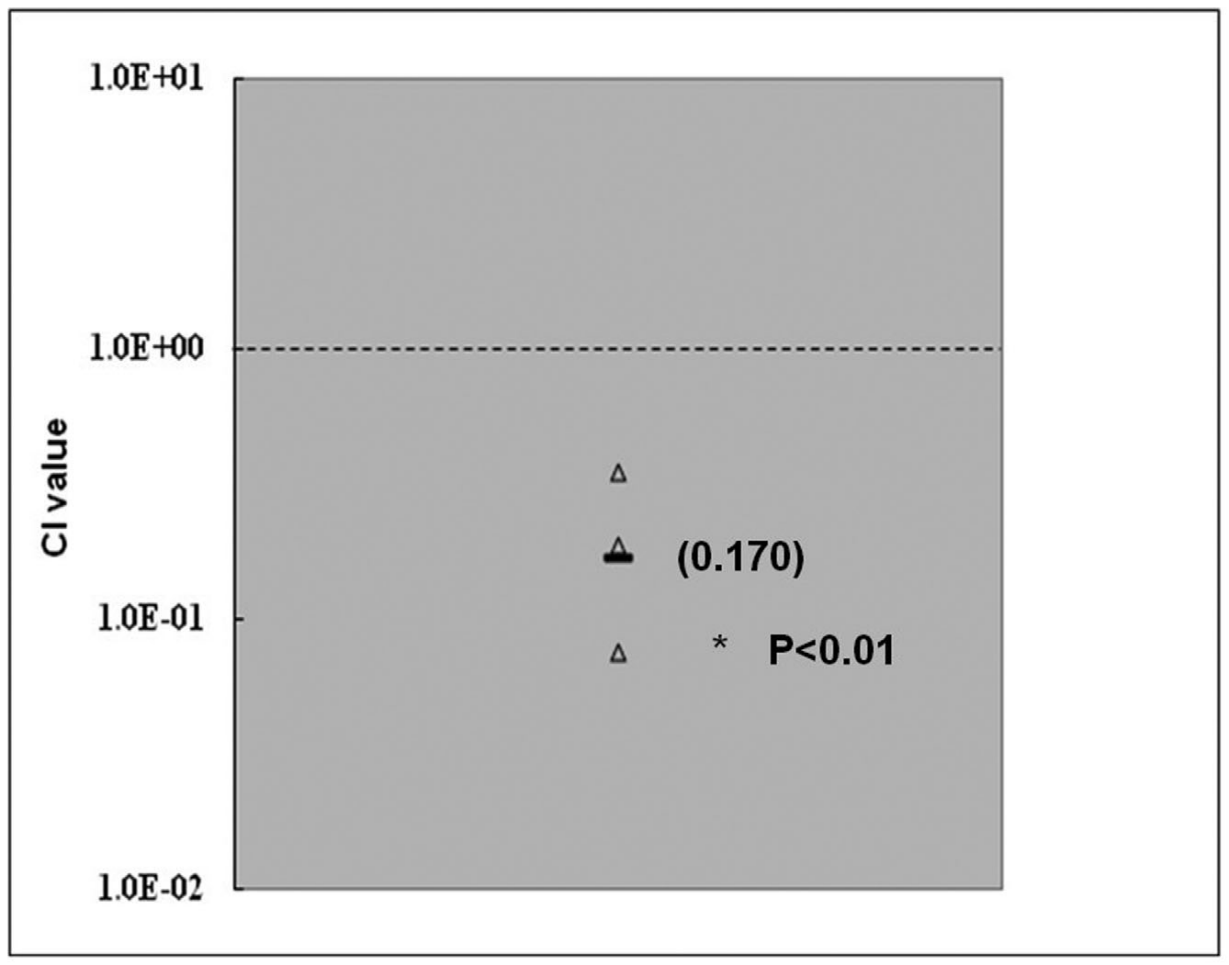
*In vivo* competitive index analyses of *ΔspxA1*. The dashed line denotes a CI value of 1, indicating equal competitiveness. Each symbol indicates the CI value derived from a single animal; solid horizontal lines indicate geometric mean values. Mean CI values from 3 rabbits tested are indicated in parentheses. Paired *t* tests were used to determine whether CI values were significantly different from 1, with α = 0.05. *P<0.01. Open triangle: *in vivo* CI analysis from 3 rabbits.

Since Δ*spxA1* was sensitive to a number of stresses (Fig. 4 and 5) and was defective in H_2_O_2_ production (Fig. 1), we examined the *in vivo* competitiveness of Δ*spxA1* by CI assays in the rabbit model of IE. Equivalent amounts of exponentially grown cells (∼10^8^ CFUs) of ΔspxA1 and JFP36, an erythromycin resistant derivative of the wild type SK36 which demonstrated the same virulence as the wild type [33] was used in this study. Strains were mixed and inoculated into rabbits previously catheterized to create sterile vegetations. Experiments were performed in triplicate. The recovery of bacterial cells in vegetations was accounted to give the competitive index. The results showed that the *in vivo* CI value for Δ*spxA1* was significantly less than 1 (with a geometric mean of 0.170) (Fig. 6). This suggested that SpxA1 was involved in IE virulence as mutagenesis of *spxA1* caused a ∼5-fold reduction in competitiveness *in vivo*.

### Genes Involved in Oxidative Stress Responses are Positively Regulated by SpxA1

To identity the SpxA1 regulon, we performed microarray studies by comparing gene expression in Δ*spxA1* and SK36. The results revealed 21 down-regulated and 39 up-regulated genes by 2-fold cutoff. Several genes involved in defense against oxidative stress (*sodA*, superoxide dismutase; *tpx*, thiol peroxidase; *nox*, water-forming NADH oxidase; *trxA*, thioredoxin and *csbD*, a general stress response protein) [23] were identified to be down-regulated in the SpxA1 null mutant (Table 1), suggesting the positive regulation of these genes by SpxA1. The expression ratios of these genes in Δ*spxA1* compared to SK36 were also validated by qPCR analysis (Table 1). The down-regulation of these stress response genes in the *spxA1* null mutant may be partially responsible for the stress sensitive phenotype of this mutant.

**Table 1:** Expression ratios of genes in *S. sanguinis* Δ*spxA1* compared with SK36 by microarray and qPCR analyses.

Gene	Name	Function	Array^a^	qPCR^b^
*ssa_2052*	*trxA*	Thioredoxin	0.240*	0.743
*ssa_1745*	*csbD*	General stressresponse protein	0.327*	0.533*
*ssa_0721*	*sodA*	Mn/Fe-dependentsuperoxide dismutase	0.387*	0.586*
*ssa_0259*	*tpx*	Thiol peroxidase	0.543	0.492*
*ssa_0391*	*spxB*	Pyruvate oxidase	ND	0.372*
*ssa_1127*	*nox*	H_2_O-forming NADHOxidase	0.490*	0.501*

a Array data are relative average levels of expression compared to expression in *S. sanguinis* SK36 from three microarray slides.

b qPCR data are ratios of relative gene copy numbers normalized by that of the house keeping gene *gyrA* relative to those of *S. sanguinis* SK36.

ND, not determined by microarray analysis.

* *P*<0.05.

### Genes Involved in H_2_O_2_ Production are Positively Regulated by SpxA1

We previously identified four genes (*ackA* (*ssa_0192*), *spxB* (*ssa_0391*), *spxR* (*ssa_1492*) and *tpk* (*ssa_2118*)) involved in H_2_O_2_ production in *S. sanguinis* by screening mutants for opaque colony appearance [28]. Recently another gene, *nox*, was also shown to be involved in H_2_O_2_ production in *S. sanguinis* SK36 (Ge *et al*., manuscript in preparation). To examine whether these other H_2_O_2_-production related genes are under the control of SxpA1, in addition to microarray studies, we also determined the transcriptional level of these H_2_O_2_-production related genes by real-time quantitative PCR (qPCR) in the wild type and Δ*spxA1*. The results showed that the expression of *spxB and nox*, which encode two oxidases ­ pyruvate oxidase and NADH oxidase respectively, decreased significantly in Δ*spxA1* in comparison with SK36 (Table 1). The expressions of *spxB* and *nox* are directly responsible for H_2_O_2_ release. This suggested that the involvement of SpxA1 in H_2_O_2_ production may be via a mechanism affecting expressions of *spxB* and *nox*.

## Discussion

In this study, we report on the characterization of the *spxA1* gene and study another *spx* gene, *spxA2*, in *S. sanguinis*. The Spx global regulator is highly conserved among low-GC Gram-positive bacteria [16]. Two Spx homologs were identified in *S. pneumoniae* [24], a double mutation of both homologs resulted in *S. pneumoniae* lethality, supporting the idea of a potential overlap in the roles of the Spx proteins [24]. Furthermore, transcriptional repression by SpxA1 also had a negative effect in the development of the X-state (competence) [24]. *S. mutans* was also reported to harbor two Spx proteins which modulate stress tolerance, survival, and virulence [23]. Our study showed that the Spx proteins were also conserved in *S. sanguinis* SK36, though its GC content is higher than other *streptococci* (43.40% compared with 35.62 to 39.72%) [4]. In *S. sanguinis*, SpxA1 and SpxA2 share high homology (44% identity in amino acids), however independent inactivation of *spxA*1 and *spxA*2 led to different phenotypes, which suggest that *spxA*1 and *spxA*2 may have different functions. The H_2_O_2_ production is an obvious phenotype of the differing phenotypes controlled by SpxA1 or SpxA2. SpxA1 controls the expressions of *spxB* and *nox* (Table 1), resulting decreased H_2_O_2_ production in the mutant (Fig. 1). While in the Δ*spxA2* mutant, though further experimental data are needed, it can be supposed that no significant difference for the expression of *spxB* and *nox* will be found because of its normal H_2_O_2_ production (Fig. 1). Because Δ*spxA2* and the double mutant Δ*spxA1A2* were significantly defective in growth (Fig. 4) and cannot be studied in the *S. mutans* inhibition experiment or our IE animal model which require equivalent growth of the studied objectives, we therefore focused on the Δ*spxA1* mutant only in subsequent studies. However, we also performed the complementation experiment for the *spxA2* mutant employing the same strategy as *spxA1*, and the results indicated that the growth and stress tolerance were restored (data not shown). We also further inactivated two of the *spxA2* downstream genes, *ssa_2243* and *ssa_2242*, respectively and examined their phenotypes. Both mutants showed no significant difference from the wild type strain concerning growth and stress tolerance (data not shown).

The *spxA2* mutant produced normal level of H_2_O_2_ (Fig. 1) that indicated the *spxA2* was not associated with production of H_2_O_2_ in *S. sanguinis*. However, it is interesting that the *spxA2* mutant was extremely sensitive to the presence of oxygen (Fig. 2A). In addition, cell survival rate of the *spxA2* mutant decreased dramatically in H_2_O_2_ treatment (Fig. 5B). H_2_O_2_ is one major contributor to oxidative damage. Our data suggested that the oxidative stress defense and repair systems may be impaired in *spxA2* mutant. The oxidative stress from the normal H_2_O_2_ production of the *spxA2* mutant may cause weak growth in the presence of oxygen.

Although inactivation of *spxA1* had no impact on the growth of Δ*spxA1* under normal temperature conditions, it did cause a significant reduction in the rate of H_2_O_2_ production (Fig. 1) and, consequently, negatively affected the competitive advantage of *S. sanguinis* towards *S. mutans* (Fig. 2). Previous reports have indicated that *S. pneumoniae* can also produce a certain amount of H_2_O_2_ [34], therefore, it would be of particular interest to determine if an ortholog of *spxA1* (*spr1262*) in *S. pneumoniae* is also involved in H_2_O_2_ production and virulence. In *S. pneumoniae,* it is thought that virulence is likely related with H_2_O_2_ production [34]. In *S. sanguinis*, Δ*spxA1* was defective in H_2_O_2_ production (Fig. 1) and at the same time, was more sensitive to exogenous H_2_O_2_ (Fig. 5B). It was reported that factors contributing to H_2_O_2_ resistance in *S. pneumoniae* include pyruvate oxidase (SpxB) [35]. In *S. sanguinis*, expression of *spxB* (*ssa_0391*) in Δ*spxA1* decreased (Table 1), suggesting that exogenous H_2_O_2_ sensitivity in Δ*spxA1* may also be related with pyruvate oxidase (SpxB). Our results also showed that inactivation of *spxA1* affected the expression of *nox* (Table 1), encoding a NADH oxidase which was related with H_2_O_2_ production (data not shown). In *S. mutans*, consistent with our results, the ortholog of *nox* in *S. mutans* which is involved in oxidative stress response, was also positively regulated by SpxA [23]. This suggested that the regulation genes of Spx protein may also be conserved to some extent.

The increasing frequency of antibiotic resistance of viridans streptococci [36], coupled with the inability to administer antibiotics in every condition underlying bacteremia, highlights the need for the advancement of preventative measures against IE, for which no vaccine exists. Previous studies have evaluated putative virulence factors for endocarditis. For example, binding to laminin, fibrin, a complex extracellular matrix preparation, as well as platelet aggregation by *S. sanguinis* are all implicated as important in causing endocarditis [9], [37]. In this study, the transcriptional regulator SpxA1, was shown to be involved in IE virulence. To our knowledge, this is the first example of *spx* gene being involved in virulence of an invasive disease such as IE. It is possible that the downstream regulatory gene, *nox*, may contribute to the involvement of SpxA1 in IE virulence because *nox* was also shown to be involved in IE virulence (Ge *et al*, manuscript in preparation); however, it is also possible that other genes under the control of SpxA1 may also contribute to the virulence in *S. sanguinis* SK36. The involvement of SpxA1 in stresses (high temperature, acidity, H_2_O_2_) tolerance may also be responsible for its involvement of IE, because the pathogenesis of IE is a multi-step process and various stresses involve during its development such as the oxidative stress in the blood. We believe there is connection between stress survival and virulence of IE. Further identification of the SpxA1 regulon that is responsible for the reduced competitiveness will provide valuable information for understanding the pathogenicity of *S. sanguinis*.

In conclusion, in this study individual gene deletions of two *spx* genes revealed their role in important phenotypes concerning growth, H_2_O_2_ production, stresses (high temperature, acidity, H_2_O_2_) tolerance and IE virulence. Further investigations on Spx proteins will provide vital information required to better understand bacterial regulatory mechanisms involved in not only IE virulence but also stress tolerance.

## Materials and Methods

### Ethics Statement

Animals were treated humanely and in compliance with all applicable federal guidelines and institutional policies. All of the procedures were approved by Virginia Commonwealth University Institutional Animal Care and Use Committee.

### Bacterial Strains and Medium

The strains used in this study are described in Table 2. *S. sanguinis* strain SK36, obtained from Dr. Mogens Kilian (University of Aarhus, Denmark), was isolated from human dental plaque [38]. *S. mutans* UA159, *S. sanguinis* SK36 and their derivatives were routinely grown in brain heart infusion broth (BHI; Difco Inc., Detroit, MI) supplemented with 1.5% (wt/vol) agar under microaerobic conditions (7.2% H_2_, 7.2% CO_2_, 79.6% N_2_, and 6% O_2_) at 37°C as previously described [13], [28], [39]. When needed, medium was supplemented with kanamycin (Sigma-Aldrich, CA) (500 µg/ml) or chloramphenicol (Sigma-Aldrich, CA) (5 µg/ml). For growth curve studies, overnight cultured bacteria were diluted 100-fold into wells of a 96-well plate containing 200 µl BHI media and the OD_450_ of each well was determined with a FLUOstar plate reader (BMG LABTECH, Offenburg, Germany) every 100 minutes under aerobic conditions. When assays were performed anaerobically, an overlay of 50 µl of sterile mineral oil was added to each well to create anaerobic conditions [23].

**Table 2:** Bacterial strains used in this study.

Strain	Phenotype or description	Source
*S. sanguinis* strains		
SK36	Human plaque isolate	(38)
Δ*spxA1*	Km^r^; Δ*spxA1*::*aphA-3*	This study
Δ*spxA1C*	Cm^r^; *spxA1+*:: *magellan2*	This study
Δ*spxA1’*	Cm^r^; Δ*spxA1*:: *magellan2*	This study
Δ*spxA2*	Km^r^; Δ*spxA2*::*aphA-3*	This study
Δ*spxA1A2*	Km^r^;Cm^r^;Δ*spxA1*::*magellan2*;Δ*spxA2*::*aphA-3*	This study
*S. mutans* strains		
UA159	Wild-type, serotype *c*	ATCC 700610
*smx_42*	Cm^r^; Δ*smu.42*:: *magellan2*	(28)

Cm, chloramphenicol; Km, kanamycin.

### Mutant Construction and Complementation

For the construction of *S. sanguinis* Δ*spxA1* and Δ*spxA2*, a PCR-based recombination method was employed as described previously [27]. Briefly, for each targeted gene, three sets of primers were designed to amplify a linear DNA fragment containing the kanamycin resistance cassette (*aphA-3*) [33] with two flanking arms of DNA upstream and downstream of the targeted gene. The linear recombinant PCR amplicon was directly transformed into *S. sanguinis* SK36 competent cells as previously described [13]. For the construction of the double mutant strain Δ*spxA1A2*, a Δ*spxA1’* strain was first constructed by replacing the *spxA1* ORF with the chloramphenicol resistance cassette from the *magellan2* mini-transposon [39]. Then using the competent cells of Δ*spxA1’* strain, the *spxA2* ORF in this strain was replaced with the kanamycin resistance cassette as described above. The mutants were confirmed by PCR and DNA sequencing analysis.

To construct the complemented strain Δ*spxA1C*, the DNA fragment containing the *spxA1* ORF followed by the chloramphenicol resistance cassette [33] was integrated via double homologous recombination into Δ*spxA1* (Table 2) to replace the kanamycin resistance cassette. A chloramphenicol*-*resistant and kanamycin-sensitive transformant was selected and confirmed by PCR analysis.

### H_2_O_2_ Release Assays

H_2_O_2_ production was quantified using an amplex red hydrogen peroxide/peroxidase kit (Invitrogen, CA) as previously described [28]. Final values are shown relative to that of the wild-type strain, SK36. Paired t-test was used for statistical analysis.

### Competition Assays

To determine the inhibitory effect of *S. sanguinis* SK36 or mutants against *S. mutans*, a competition assay on agar plates was employed as described previously [28]. Growth inhibition was evaluated based on the distance of the inhibition zone between the edges of both colonies.

Competition assays in liquid media was performed as described previously [28]. Cells of *S. sanguinis* SK36 or Δ*spxA1* and *S. mutans smx_42*, a chloramphenicol-resistant derivative of *S. mutans* (Table 2), were grown in BHI medium overnight and adjusted to the same OD_660_. *S. sanguinis* or Δ*spxA1* (3 µl of each) and *S. mutans smx_42* (3 µl) were mixed with 200 µl fresh BHI medium in 96-well microtiter plate in triplicate. The cells were incubated overnight and then dispersed by vigorous pipetting and serial dilutions were plated on BHI agar plates supplemented with chloramphenicol. Assays were performed in triplicate and CFU counts were determined. Paired t-test was used for statistical analysis.

### Acid Tolerance Assays

Acid tolerance experiments were performed as described previously [23] with the following modifications. Briefly, overnight cultures of *S. sanguinis* SK36 or mutants were diluted 100-fold into fresh BHI medium and cultured for 5 h under microaerobic conditions at 37°C. For each strain, three biological replicates were included. The cells were then harvested and washed once with one culture volume of 0.1 M glycine buffer (pH 7.0) and resuspended to give an OD_660_ of ∼2.0. Samples were centrifuged again and resuspended in the same volume of 0.1 M glycine buffer (pH 3.0) for 60 min. Aliquots were then serially diluted, plated on BHI agar, and incubated for 48 h before colonies were counted. Survival after treatment was determined as the percentage of the wild type, and the statistical significance of differences with SK36 were determined by paired t-test analysis.

### H_2_O_2_ Sensitivity Assays

Exponentially growing cultures of *S. sanguinis* strains were prepared, washed and adjusted to OD660∼2.0 as described above. Triplicate 100 µl aliquots of each culture were centrifuged and resuspended in fresh BHI medium containing 20 mM H_2_O_2_ that had been diluted from a 30% (9.8 M) stock solution (Invitrogen), followed by incubation at 37°C for 60 min [35]. Serial dilutions from each sample were then plated onto BHI agar, and colonies were counted after 48 h incubation. The percentage survival was calculated by dividing the CFU of cultures after exposure to H_2_O_2_ by the CFU of a control tube without H_2_O_2_.

### Competitive Index (CI) Assays

A CI assay for a rabbit model of IE was used to evaluate the competitiveness of *ΔspxA1* as previously described [40], [41]. Briefly, for the preparation of the CI inoculum, overnight cultures of JFP36 [33], an erythromycin-resistant derivative of the wild type SK36 which demonstrated the same virulence as the wild type [33], and the mutant *ΔspxA1* grown in BHI were diluted 10-fold into 14 ml pre-warmed BHI for an additional 3 h of growth at 37°C. Cells were then washed and suspended in phosphate-buffered saline (PBS) to give an OD_660_ of 0.8, corresponding to ∼10^8^ bacteria/0.5 ml. Equal volumes of *ΔspxA1* and JFP36 cells were then combined to make an inoculum of 1.0 ml. This inoculum was sonicated at 50% power for 1.5 min in a titanium cup adaptor (BioLogics Inc., Manassas, VA). An inoculum of 0.5 ml was then inoculated into each rabbit into which cardiac catheters had previously been inserted to create sterile vegetations. Experiments were performed in triplicate. The day after inoculation, rabbits were sacrificed and heart valve vegetations were collected. The bacterial cells in vegetations were homogenized, sonicated as per the inoculum, serially diluted and spread-plated onto BHI plates supplemented with erythromycin or kanamycin for enumeration. The CI was determined as the ΔspxA1:JFP36 ratio of the output CFUs divided by the ΔspxA1:JFP36 ratio of the inoculum. Paired t-test was used to determine whether CI values were significantly different from 1, with α = 0.05.

### Microarray Analysis

Total RNA from each of three independent samples of *S. sanguinis* SK36 and Δ*spxA1* was prepared from cells growing exponentially in BHI medium under microaerobic conditions for 5 h (OD_660_ ∼0.8). Cells were lysed after lysozyme treatment and mechanical disruption using FastPrep® lysing matrix B (Qbiogene, CA). RNA was isolated with an RNeasy mini kit (Qiagen, Hilden, Germany). DNA was removed from the RNeasy mini kit column by DNase I treatment. Total RNA was quantified using a NanoDrop® ND 1000 Spectrophotometer. Spotted microarray slides were obtained from the Pathogen Functional Genomics Resource Center at J. Craig Venter Institute (JCVI, MD). The microarray was performed according to the manufacturer’s protocol (http://pfgrc.jcvi.org/index.php/microarray/protocols.html). Hybridization was performed at 42°C for 16–20 h. Microarray slides were scanned on a GenePix 4000A scanner. Images were analyzed using the program Spotfinder (v321win). Data were LOWESS normalized and background subtracted using Midas (v2.20). Processed red signal/processed green signal ratios were calculated. Microarray data has been deposited into the NCBI Gene Expression Omnibus (GEO) with access number GSE34203.

### qRT-PCR Analysis

Quantitative RT-PCR (qRT-PCR) was performed as described previously [28]. Total RNA was prepared as described above. First-strand cDNA synthesis was performed using SuperScript® III Reverse Transcriptase (200 U/µl, Invitrogen). Reactions lacking reverse transcriptase were prepared in parallel as negative controls for possible DNA contamination. First-strand cDNA from each reaction was subjected to eighty-fold dilutions, and 2 µl of each dilution was used as template for each PCR reaction. PCR was performed in reactions containing 5 µl of SYBR® Green PCR Master Mix (Applied Biosystems), and 1 µl of each PCR primer at 2 mM using the ABI 7500 Fast Real-Time PCR system. Data was collected and statistically analyzed from triplicate biological samples. The amount of relative gene transcript was normalized with that of *gyrA* in each sample. Data are reported as the percentage of the amount of normalized transcript from the wild type SK36.
